# Dynamic Hip Screw for the Treatment of Femoral Neck Fractures: A Prospective Study with 96 Patients

**DOI:** 10.1155/2014/257871

**Published:** 2014-04-24

**Authors:** Carlos Roberto Schwartsmann, Lucas Senger Jacobus, Leandro de Freitas Spinelli, Leonardo Carbonera Boschin, Ramiro Zilles Gonçalves, Anthony Kerbes Yépez, Rodrigo Py Gonçalves Barreto, Marcelo Faria Silva

**Affiliations:** ^1^Orthopaedics and Traumatology, Federal University of Health Sciences of Porto Alegre and Santa Casa de Misericordia of Porto Alegre, Rua Leopoldo Bier, 825/cj 403, 90620-100 Porto Alegre, RS, Brazil; ^2^Department of Orthopaedics and Traumatology/Pediatrics, Santa Casa de Misericordia of Porto Alegre, Avenida Independência, 155, 90035-074 Porto Alegre, RS, Brazil; ^3^Department of Orthopaedics and Traumatology/Hip Surgery, Santa Clara Hospital, Santa Casa de Misericordia of Porto Alegre, Reception 8, Rua Professor Annes Dias, 135, 90020-000 Porto Alegre, RS, Brazil; ^4^Department of Physical Therapy, Santa Clara Hospital, Santa Casa de Misericordia of Porto Alegre, 2nd Floor, Rua Professor Annes Dias, 135, 90020-000 Porto Alegre, RS, Brazil; ^5^Department of Physical Therapy, Federal University of Health Sciences of Porto Alegre, Rua Sarmento Leite, 245, 90050-170 Porto Alegre, RS, Brazil

## Abstract

*Objectives*. To study the correlation between avascular necrosis and the demographics, time elapsed from fracture to surgery, quality of reduction, Garden classification, and the position of the screw following use of the dynamic hip screw (DHS) in the treatment of subcapital neck fractures. *Methods*. A prospective study of 96 patients with subcapital neck fractures was carried out in a faculty hospital. Patients underwent surgery with closed reduction and internal fixation with DHS. *Results*. There were 58% male and 42% female patients, with a mean age of 53 years (+/−14). In terms of Garden classification, 60% were Garden IV, 26% were Garden III, and 14% were Garden II. Nonunion was observed in three cases (3%) and was treated with valgus intertrochanteric osteotomy, in all cases leading to successful healing. Avascular necrosis was observed in 16% of patients. The positioning of the screw into the femoral head showed a significant correlation with necrosis. *Conclusions*. The incidence of necrosis in patients under the age of 50 years is twice as high as that in older patients. Displacement is a predictive factor regarding osteonecrosis and is associated with a high and anterior position of the screw in the femoral head. Level II of evidence. Study Type: therapeutic study.

## 1. Introduction

Surgical management of displaced subcapital fractures of the femoral neck continues to be challenging. Internal fixation, hemiarthroplasty, and total hip replacement could be considered as appropriate solutions.

For internal fixation, most orthopaedic surgeons choose either a dynamic hip screw (DHS) or multiple cannulated screws (MCS). Osteosynthesis with MCS fixation is a less invasive technique and reduces blood loss and soft tissue stripping [1–3]. With the use of DHS the screw-plate system achieves a more stable condition. Deneka et al. [4] published a biomechanical comparison of internal fixation techniques for the treatment of unstable basicervical femoral neck fractures. The results support the use of DHS. Its disadvantages are large skin incisions, more extensive soft tissue dissection, a greater need for blood transfusion, and a longer stay in hospital.

In a cross-sectional survey using a regressive analysis, Bhandari et al. [5] suggested that surgeons in Europe were more likely to indicate a DHS device over MCS than North American surgeons. Krastman et al. [3] defended the use of only two cannulated screws in nondisplaced femoral neck fractures but suggested DHS, as a more stable implant, for Garden III-IV fractures. Lee et al. [6], after reviewing 90 fractures, concluded that DHS showed a trend for an increased rate of overall success in elderly patients with nondisplaced femoral neck fracture compared with MCS.

There are few published reports focusing on DHS in the treatment of femoral intracapsular displaced neck fractures. Parker and Blundell [7] analysed the use of these implants for internal fixation. They reviewed 25 randomized trials and concluded that most studies have had an insufficient number of subjects to permit a valid comparison. Yih-Shiunn et al. [2] found overall failure in 15.9% of cases using MCS and in 2.5% of cases using DHS. Chen et al. [8], using DHS in extracapsular basicervical neck fractures, achieved union in 97.5% of their patients, with no cases of avascular necrosis and 1.7% of nonunion. Osteosynthesis not only has the potential to offer normal hip function after fracture consolidation but also has a relatively high rate of failure and complications in the form of nonunion, avascular necrosis, redisplacement, and so forth.

The purpose of the present study was to correlate the incidence of avascular necrosis following treatment with the DHS with patient demographics, time elapsed from the fracture to surgery, quality of reduction, Garden's classification [9], and the position of the screw in the head.

## 2. Materials and Methods

The present study prospectively evaluated 96 patients who had subcapital fractures of the femoral neck and were assigned to close reduction and internal fixation with DHS. Patients were operated on from 2000 to 2006. The inclusion criterion was subcapital fractures of the femoral neck and not tumoral ones, whereas exclusion criteria were bad quality X-rays pre- or postoperatively, more than a week since fracture, comminuted fractures, dislocated fractures classified as Garden III and IV in patients older than 75 years, rheumatoid arthritis and metabolic diseases (including osteoporosis Singh stage III or less), and incomplete records. We prospectively followed 128 patients, but 32 cases were excluded from the final analysis according to the exclusion criteria. The remaining 96 fractures were available for evaluation of functional results and complications. Four patients died but were still included, because they died 3 years, 4 years and 6 months, 8 years, and 7 years, respectively, after the onset of fracture.

All surgeries were performed by closed reduction, on a standard fracture table assisted by fluoroscopy. Minimal traction and rotation were applied in the first instance. If the fracture was incompletely reduced, small incremental increases in both traction and internal rotation were subsequently performed, checking the position after each adjustment [10]. A standard internal fixation with a 135° DHS was used, varying the number of screws and length of the plate as required.

Reduction was judged on both the anterior-posterior (AP) and lateral views. The junction of the convex femoral head and neck should produce an S-shaped curve in all planes [11]. A valgus reduction is inherently more stable, whereas a varus reduction is associated with a much higher risk of fixation failure [12]. What constitutes an acceptable reduction is debatable, and Arnold [13] recommended that there should be less than 20 degrees of posterior angulation to minimize the risk of fixation failure. Garden [14] described an alignment index to measure the quality of reduction, which was used in the present work.

Demographics, age at the onset of fracture, trauma mechanism, Garden's classification [9], associated fractures, time elapsed to surgery, American Society of Anaesthesiologists' (ASA) [15] criteria, Baumgaertner index, and hospital discharge were evaluated. The tip-apex distance (TAD) of all lag screws was measured, as described by Baumgaertner et al. [16], and the position of the screw in the head was classified in the anterior-posterior projection as high, middle, or low. The same was done in the lateral projection, classifying the position as anterior, central, or posterior. The criterion for good reduction was normal or slightly valgus alignment. Clinical results were assessed using the D' Aubigné and Postel score [17].

The accepted definition of union was the development of a well-established trabecular pattern across the fracture site within 6 months following the date of injury. Avascular necrosis of the femoral head was diagnosed based on progressive pain with the classic mottled appearance, increasing radiodensity, segmental collapse, and degenerative changes. The Ficat staging system was used to evaluate the patients.

The Fisher method of statistical analysis was used to correlate all possible positions of the screw in the head with avascular necrosis.

## 3. Results

Table 1 presents the characteristics of the 96 patients. There were 56 (58%) males and 40 (42%) females with an overall mean age at the onset of fracture of 53 years (±14), ranging from 18 to 70 years. Seventy-eight patients (81%) had experienced a simple fall. Sixteen (16%) had fallen from height and three (3%) were victims of automobile traffic trauma. The mean follow-up was 64.6 months.

With respect to Garden's classification for femoral neck fractures, 58 (60%) were identified as Garden IV, 25 (26%) were Garden III, and 13 (14%) were Garden II. Fifty patients (52%) had fractures of the right hip and 46 (48%) of the left one. Eleven patients had associated fractures: three distal radius fractures, three rib fractures, two ankle fractures, two proximal humeral fractures, and one tibial shaft fracture.

Only one (1%) patient was operated on in the first 24 hours. Fifty-five (57%) patients were operated on between 24 to 72 hours after fracture. Forty (42%) were operated on after 72 hours. When considering clinical conditions, 68 (71%) were considered ASA I (normal healthy) and 28 (29%) were ASA II (mild systemic disease).

The DHS plate was fixed with two screws in five patients (5%), while three screws were used in 61 patients (64%), and four screws in 30 patients (31%). Good reduction was obtained in 94 (98%) of the patients, which means normal or slightly valgus reduction. The average hospital discharge was 9 days.

The greatest penetration into the femoral head for the lag screw of the DHS was 66 mm, whereas the least penetration was 31 mm, with an average of 42.3 mm. The mean TAD was 16 mm (ranging from 11 to 24 mm).

A satisfactory union was achieved in 80 patients, with a failure rate of about 20%. Three patients failed to show union after 6 months (3%) and presented criteria justifying surgical reintervention. In these cases, the radiographs showed no radiological signs of consolidation, with resorption of the fracture, pain, and synthesis failure. All of these nonunions were treated with a valgus intertrochanteric osteotomy, all of them achieving successful healing.

Sixteen cases of avascular necrosis were observed (16%), ten of which were classified as Ficat 3 and six as Ficat 4. Most of our patients (58%; 56/96) were operated on in the first 72 hours. However, a substantial number was operated on after 72 hours (42%; 40/96). The percentage of osteonecrosis in the latter group was 23% (9/40), being almost double that of the former (13%; 7/56). The average age of these patients was 45 years. The latest diagnosis was 5.6 years after the fracture. Four fractures healed with shortening of the femoral neck of less than 15 mm.

**Figure 1:**
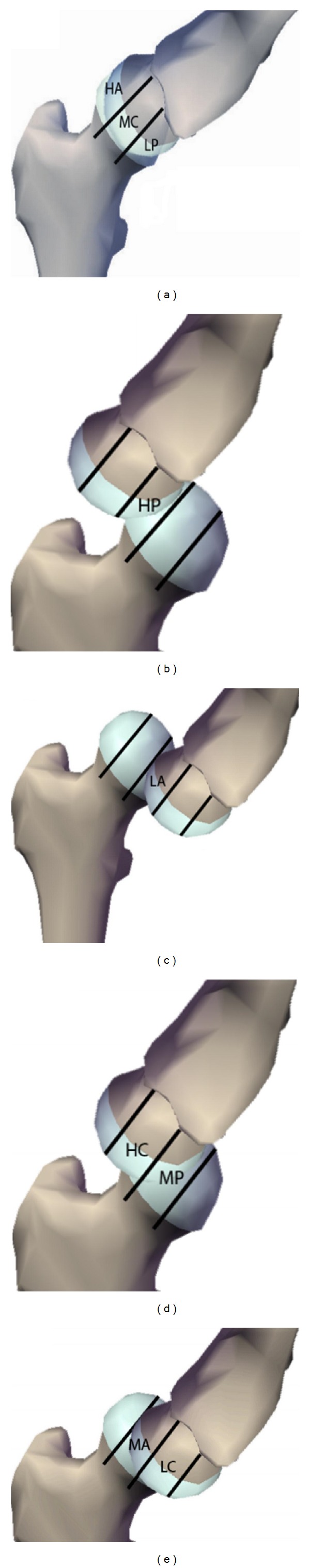
Avascular necrosis versus screw placement on the femoral head. H = high; M = middle; L = low; A = anterior; P = posterior; C = centre.

We also analysed the screw position based on all nine possibilities presented in Figure 1 for the AP and lateral projections (see also Table 2) and correlated them with osteonecrosis. The most frequent position was middle (AP) and central (lateral view), as observed in 47 patients (49%), with four cases of osteonecrosis (9%; 4/47). The combination high (AP) and anterior (lateral) was observed in eight fractures (8%) and five of these patients developed osteonecrosis (63%; 5/8). This was the only statistically significant association between screw placement and osteonecrosis (*p* = 0.0029, using Fisher's correction).

Regarding the D' Aubigné and Postel score, sixty patients (63%) scored 18 points, 18 (19%) scored 17 points, and 18 (19%) scored less than 17 points.

We correlated age, gender, side, time elapsed until surgery, Garden's classification, and quality of reduction with avascular necrosis and no statistical differences were found, but when we analysed the position of the screw in the femoral head we found a significant correlation between necrosis and the high, anterior position (*p* = 0.003).

## 4. Discussion

The treatment of displaced femoral neck fractures has been debated for many years. The main question during decision making is whether to fix or replace the femoral neck. Many recently published papers have shown that a primary total hip replacement is superior to internal fixation for the treatment of displaced femoral neck fractures when performed in a relatively healthy and mentally competent elderly patient [5, 7, 18–20]. However, the optimal treatment for a young or adult patient under 70 years old is controversial, as the younger the patient is, the more the orthopaedic surgeon is obliged to pursue internal fixation.

There are many factors that could influence the decision: the preinjury functional status regarding gait, mental ability, and habitat, but the most important consideration is probably the difference between chronological and biological age. Criticisms against internal fixation are due to its association with high rates of failure due to loss of fixation, osteonecrosis, and nonunion. Nevertheless, when a femoral head heals over the neck, the patient has the chance of regaining his physiologically normal hip.

Lu-Yao et al. [21] published a meta-analysis of 106 published reports and concluded that the rate of loss of fixation or reduction after open reduction and internal fixation (ORIF) is about 16% (9–27%), which is significantly higher than the risk of dislocation after hemiarthroplasty (2%) or total hip replacement. Tronzo [22] identified more than 100 different available implants for ORIF of femoral neck fractures. However, if a surgeon chooses ORIF, he basically must decide between two consecrated techniques: multiple cannulated screws (MCS) or a dynamic hip screw (DHS).

Several studies have attempted to identify predictive factors of failure in femoral neck fracture treatment. There is little agreement among these studies regarding which fractures are more likely to fail because they analysed both displaced and nondisplaced fractures, different clinical and radiological factors, different implants for internal fixation, different weight-bearing times, and so forth.

The results of osteosynthesis in young patients are debatable by presenting a considerable complication rate. However, there is little doubt that the main complication is the occurrence of osteonecrosis. Many variables have been hypothesized to be associated with this complication after femoral neck fractures. The literature does not support differences regarding gender, but high rates of nonunion and avascular necrosis are more common in young adult patients. Various explanations have been elaborated, including high energy trauma and its correlation with dislocated fractures in the young adult. The rate of osteonecrosis ranges from 12% to 86% [23–27]. Gautam et al. [26], while operating on 25 young adults on an ordinary table using the traditional Watson-Jones approach, described three cases of osteonecrosis (12%). Protzman and Burkhalter [23], reviewing 22 fractures in young patients aged under 40 years, found 86% of necrosis. We divided our cases according to age into two main groups: under 50 years (46 fractures) and over 50 years (50 fractures). The incidence of osteonecrosis was 22% (10/46) in the first group and 12% (6/50) in the second one. No statistical difference was found.

Another topic of discussion is the amount of initial fracture displacement. The most useful classification was proposed by Garden [9]. Basically, the author divided the fractures into not displaced (Garden I and II) or displaced (Garden III and IV). In our study, only 13 fractures were considered nondisplaced (14%) and 83 were considered displaced (86%). We did not find any osteonecrosis in the first group, while there was an incidence of 19% (16/83) in the second group (*p* = 0.12).

Conn and Parker [28], when evaluating 375 nondisplaced fractures, observed necrosis in 4% (15/375). Yih-Shiunn et al. [2] reviewed 84 cases of nondisplaced fractures and found an incidence of about 10% (8/84). Haidukewych [25] found 14% (3/22) and Nikolopoulos et al. [29] found 19.5% (9/46).

When only displaced fractures are taken into consideration, this complication is more frequent. In an extensive meta-analysis, Lu-Yao et al. [21] found a 16% rate of osteonecrosis, and Blomfeldt et al. [19] recorded 19% of cases with necrosis after 48 months. Majerníček et al. [30] observed 13.4% (9/64) after a minimum of 5 years of follow-up. Haidukewych [25] found 27% (14/51), and Nikolopoulos et al. [29] found 39.4% in displaced fractures (15 out of 38) after a mean follow-up of 4.7 years. Kaplan et al. [31] recently performed a study comparing open and closed reduction with internal fixation. Avascular necrosis was more common in displaced fractures (30.3%; 10/33).

Another controversial issue is the timing of surgery. Barnes et al. [32], in their historical paper, describe a long-term follow-up of 1503 subcapital fractures and conclude that the mortality rate increased when operation was delayed beyond 3 days following injury, but no significant difference was found in necrosis or late segmental collapse when delaying the operation up to 1 week. Most of our patients were operated on in the first 72 hours (58%; 56/96). However, a substantial number was operated on after 72 hours: 42% (40/96). The percentage of osteonecrosis in the latter group was 23% (9/40), being almost double that of the former: 13% (7/56). This suggests that it could be worse to fix the fracture more than 72 hours after the fracture's onset, but no statistical difference was found between operating earlier or later in terms of necrosis (*p* = 0.41).

Advocates of early surgery suggest that prompt reduction can produce an “unkinking” of the proximal femoral vessels, thus leading to intracapsular decompression, restoring the blood flow to the femoral head and minimizing the risk of necrosis [33, 34]. Other studies confirm that early surgery may decrease the rate of femoral head osteonecrosis [35–38]. On the contrary, several studies have reported no difference in the rate of osteonecrosis with more than a 24-hour delay.

Upadhyay et al. [39] performed a prospective and randomized study of 102 patients, comparing open and closed reduction with internal fixation. Time to surgery did not affect the development of osteonecrosis. In a retrospective review of 73 femoral neck fractures Haidukewych et al. [40] reported the same outcome. He found a rate of osteonecrosis of 23%. He reported that 25% (17/73) of femoral neck fractures that were treated within 24 hours of diagnosis developed osteonecrosis. Twenty percent of the fractures that were internally fixed after 24 hours developed the same complication (4/20).

The quality of fracture reduction or postreduction malalignment is another topic of discussion. Most authors agree that the best position is anatomical reduction or a slight valgus [6, 32, 38]. In our study, of the 96 fractures we considered as a good quality reduction, necrosis occurred in 16 cases (16%). Only two patients had what we considered a slight varus reduction. Neither of these developed osteonecrosis.

The controversy about screw position in the femoral head has remained unresolved until today. The main point of discussion concerns central versus posterior-inferior screw placement. There is a consensus that the anterior-superior position should be avoided [8, 16, 32]. Barnes et al. [32] were probably the first to call attention to the fact that a nail or screw placed too superior and anterior in the femoral head was associated with a considerable failure rate in women (37% in Garden III and 52% in Garden IV). However, the present research demonstrates that the incidence of osteonecrosis is correlated with the position of the screw in the femoral head. Since Barnes et al.'s study [32], there has been no paper in the literature regarding the position of the screw in relation to avascular necrosis. They were the first to call attention to the association between the anterior-superior position of the screw and worse results. The authors recognize that the present study has a nonrandomized nature, absence of control group, and a small number of patients, but we realize that our findings, although preliminary, are similar in relation to necrosis when comparing to the literature.

## 5. Conclusions

No statistically significant association was found between gender, time elapsed to surgery, quality of reduction and fracture displacement, and the onset of avascular necrosis of the femoral head. The incidence of osteonecrosis in patients under 50 years was twofold higher than in patients over 50 years of age, but this difference was not statistically significant. The fracture's displacement is a predictive factor regarding osteonecrosis. The incidence of osteonecrosis was associated with the “high and anterior” position of the screw in the femoral head.

## Figures and Tables

**Table 1:** Demographics and characteristics of evaluated cases.

Characteristic	Number	(%)	Osteonecrosis	(%)
Sex				
Male	56	58	9	9
Female	40	42	7	7
Age group				
18–40	21	22	4	4
41–50	25	26	6	6
51–60	18	19	4	4
61–70	32	33	2	2
Garden grade				
II	13	14	—	—
III	25	26	4	4
IV	58	60	12	13
Time of surgery				
<24 hours	1	1	—	—
24–72 hours	55	57	7	7
>72 hours	40	42	9	9
Reduction (AP view)				
Neutral	61	64	11	11
Valgus	33	34	5	5
Varus	2	2	—	—
Reduction (lateral view)				
Neutral	69	72	10	10
Anterior	19	20	4	4
Posterior	8	8	2	2

**Table 2:** Avascular necrosis versus screw placement on the femoral head.

Screw position in the femoral head	Number	(%)	Necrosis	(%)	*p*
H-A	8	8	5	5	0.003
H-C	8	8	3	3	0.126
H-P	1	1	0	—	—
M-A	3	3	1	1	0.425
M-C	47	49	4	4	0.054
M-P	5	5	1	1	0.607
L-A	2	2	1	1	0.307
L-C	11	12	1	1	0.684
L-P	11	12	0	—	—

Total	96	100	16	16	

H: high; M: middle; L: low; A: anterior; P: posterior; C: central.
